# Inhibitor Eradication in Postpartum Acquired Haemophilia A: Real‐Life Case Series and Literature Review

**DOI:** 10.1111/hae.70020

**Published:** 2025-03-07

**Authors:** Gaetano Giuffrida, Uros Markovic, Stephanie Grasso, Andrea Duminuco, Gabriella Santuccio, Manlio Fazio, Giuliana Giunta, Lara Gullo, Chiara Sorbello, Sara Frazzetto, Salvatore La Penta, Mariasanta Napolitano, Gianluca Sottilotta, Francesco Di Raimondo, Gabriele Sapuppo

**Affiliations:** ^1^ Division of Hematology and Bone Marrow Transplantation Azienda Ospedaliero‐Universitaria Policlinico G. Rodolico – San Marco Catania Italy; ^2^ Regional Reference Center for the Prevention Diagnosis, and Treatment of Rare Congenital Disease in Children and Adult Patients Catania Italy; ^3^ Rare Hematological Diseases Unit Catania Italy; ^4^ Postgraduate School of Hematology University of Catania Catania Italy; ^5^ Department of Health Promotion, Mother and Child Care Internal Medicine and Medical Specialties (ProMISE) University of Palermo and Policlinico Paolo Giaccone, Unit of Hematology Palermo Italy; ^6^ Hemophilia Centre Grande Ospedale Metropolitano Reggio Calabria Italy

**Keywords:** acquired haemophilia A, bleeding disorder, immunosuppressive therapy, inhibitor, postpartum

## Abstract

**Background:**

Acquired haemophilia A (AHA) is a rare and severe bleeding disorder generally associated with pregnancy or aging. Spontaneous remission and prompt inhibitor eradication are described more frequently in postpartum cases. We evaluated retrospectively 15 postpartum AHA cases between 2007 and 2023 in order to evaluate response in terms of inhibitor eradication.

**Results:**

The median age at diagnosis was 31 years (range 24–38). All patients reported bleeding at presentation after a median period of 40.6 days following delivery (range 2–180 days). The median FVIII level was 4.4% (range 0%–12.8%), with a median FVIII‐inhibitor titer of 35 BU (range 2–156). The most severe bleeding symptoms were metrorrhagia and genital bleeding in nine patients (60%), and one patient had an important muscular haematoma. Two patients underwent hysterectomy before diagnosis due to severe bleeding. All patients required anti‐haemorrhagic therapy with a median duration of 8 days (range 1–28 days): 60% (9/15) with eptacog alfa, two with an activated prothrombin complex concentrate, and in combination in four cases. The immunosuppressive treatment was corticosteroids alone in eight patients (53%), cyclophosphamide or azathioprine in combination with corticosteroids in four, while rituximab was used in two cases following traditional immunosuppressive therapy. After a median period of 28 days (range 10–210 days), the anti‐FVIII inhibitor was eradicated with normalisation of coagulation in all but one patient. However, immunosuppressive therapy, including tapering, had a median duration of 2.3 months (range 1–23 months). At the time of data censoring, all patients were alive and well at the last follow‐up with no significant adverse events.

**Summary/Conclusion:**

Notwithstanding that postpartum AHA has been reported to have a high rate of spontaneous remission, nearly half of this series experienced inhibitor eradication more than 1 month after disease onset and using immunosuppressive treatment for more than 2 months, with additional drugs being used in more than 40% of them, thus showing difficulties in disease remission in this postpartum AHA subpopulation.

## 1 Introduction

Acquired haemophilia A (AHA) is an autoimmune disease caused by the spontaneous production of neutralising immunoglobulin G autoantibodies directed against functional epitopes of endogenous coagulation factor VIII (FVIII). In the presence of an inhibitor, factor VIII activity demonstrates a rapid initial inactivation phase followed by a slower equilibrium phase [1].

AHA is predominantly a disease of the elderly (median age 64–78 years) and is idiopathic in most cases. While older patients are predominantly males, younger patients are more frequently females. The principal risk factor for the occurrence of AHA in young women is pregnancy and the majority of cases are diagnosed after delivery (1%–5%) [2]. Even though peri/postpartum AHA is predominantly diagnosed in primigravidae, it can also occur in later pregnancies [3]. The most frequently reported bleeding patterns are of similar severity and distribution in the postpartum cases as in other AHA cases (cutaneous, muscular and joint), with the addition of significant obstetrical bleeding (uterine/vaginal) [4, 5]. Haemostatic management is comparable to that of other AHA patients, and bypassing agents should be administered according to guidelines, availability and personal experience. Regarding immunosuppressive therapy, corticosteroids alone should be considered as the first‐line therapy in AHA, although spontaneous remission has been described in the literature [6, 7, 8]. Given the relative rarity of postpartum AHA, we chose to retrospectively evaluate 15 patients diagnosed and treated in three haematology divisions in Southern Italy and emphasise some differences in comparison to the data reported in the literature.

## 2 Patients and Methods

This retrospective multi‐centre case series included 15 postpartum AHA cases referred to three haematology divisions in Sicily (Catania and Palermo) and Calabria (Reggio Calabria) between 2007 and 2023. The clinical data was collected starting from AHA diagnosis during haemostatic and immunosuppressive treatment until disease resolution. Informed consent was obtained from each patient, and the study was conducted in accordance with the International Conference on Harmonization Guidelines on Good Clinical Practice and the principles of the Declaration of Helsinki.

AHA was diagnosed based on low FVIII plasma levels together with the presence of FVIII inhibitor using the Bethesda assay in concomitance with postpartum bleeding. The study aimed to retrospectively evaluate using health records the baseline characteristics of AHA patients (previous pregnancies and abortions, presence of autoimmune disease), clinical signs and symptoms, laboratory values, the anti‐haemorrhagic and immunosuppressive treatments used and response time in terms of increase of factor VIII and inhibitor eradication.

Complete remission was defined as normal FVIII and undetectable inhibitor titer using the Bethesda assay following the dose reduction or discontinuation of the immunosuppressive therapy. Partial remission was defined as FVIII levels greater than 50 UI/dL and no bleeding for at least 24 h after stopping all haemostatic treatments. If the inhibitor titer was not available, the patient was defined as ‘at least partial remission’ and considered as a partial remission.

Qualitative results were summarised in numbers and percentages. Descriptive statistics were reported as medians and ranges. All calculations and graphs were performed using MedCalc Statistical Software, version 13.0.6 (MedCalc Software, Ostend, Belgium).

## 3 Results

### 3.1 General Clinical Data

The median age at diagnosis was 31 years (range 24–38). All the 15 patients reported at presentation bleeding episodes after a median period of 40.6 days following delivery (range 2–180 days), 11 of them (73%) being diagnosed during the first postpartum month. Four patients with a delayed onset were diagnosed 2‐, 4‐, 4‐, and 6‐months from delivery. Seven patients were primiparae, three of them had a history of abortion. Five patients had pre‐existing autoimmune disorders: two had hypothyroidism, with systemic lupus erythematous, rheumatoid arthritis and psoriatic arthritis reported in single patients. Moreover, antinuclear antibody tested positive in one patient without clinical evidence of other autoimmune disease. The median level of FVIII was 4.4% (range 0%–12.8%), with a median FVIII‐inhibitor titer of 35 BU (range 2–156 BU) using the Bethesda assay. The most severe bleeding symptoms were metrorrhagia and genital tract bleeding in nine patients (60%), one patient developed a vast right calf muscular haematoma, measuring 7 cm in diameter and with a 3 g/dL drop of haemoglobin level to 8.5 g/dL, while cutaneous bleeding occurred in one‐third of the cases (Table 1). Two patients had undergone hysterectomy due to severe bleeding before diagnosis. None of the babies delivered experienced bleeding in the post‐partum period and during the first months after delivery.

**TABLE 1:** Baseline characteristics at diagnosis of patients with AHA.

	*N* patients (%)
Patients	15
Age, years (range)	31 (24–38)
Time from delivery, days (range)	40.6 (1–80)
>30 days	4 (27)
<30 days	11 (73)
*N* of pregnancy	
First pregnancy	7 (47)
Second or more pregnancies	8 (53)
Previous abortions	3 (20)
Autoimmune disorders	5 (33)
Median FVIII level at diagnosis, % (range)	4.4 (0.3–12.8)
Median inhibitor titer at diagnosis, BU/mL (range)	35 (1.9–156)
Major bleeding site	
Vaginal‐uterine	9 (60)
Muscular	1 (7)
Cutaneous	5 (33)

### 3.2 Haemostatic and Immunosuppressive Treatment

**FIGURE 1:**
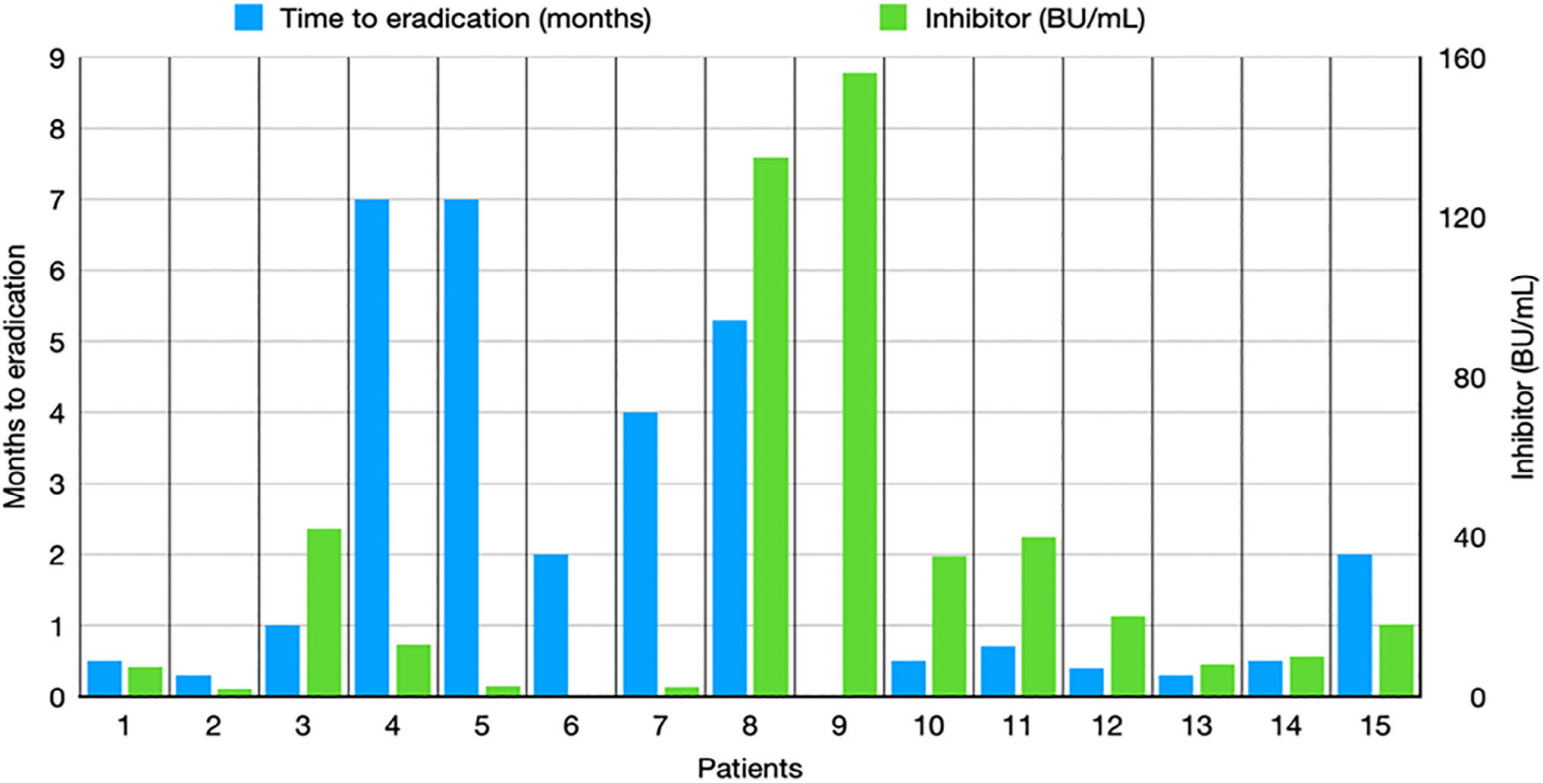
Laboratory characteristics (FVIII levels, inhibitor titer) and time required for inhibitor eradication in women with post‐partum AHA.

**FIGURE 2:**
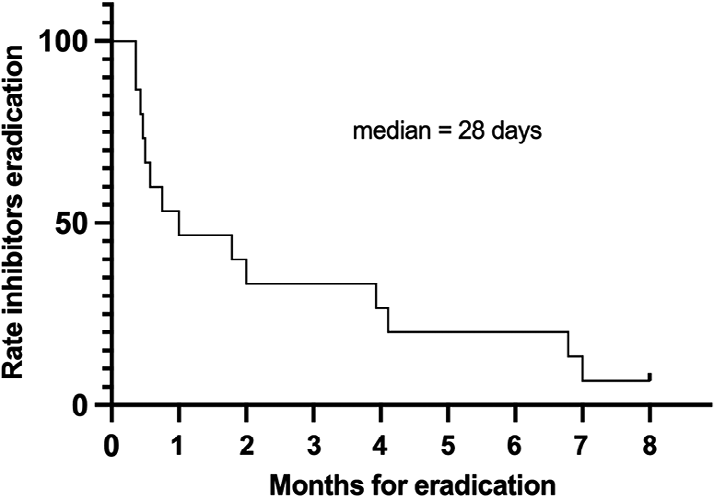
Time to obtain FVIII‐inhibitor eradication in women with AHA (*n* = 15).

All patients started haemostatic and immunosuppressive treatment on the same day of the diagnosis. All received anti‐haemorrhagic therapy with a median duration of 8 days (range 1–28 days): 11 of them (73%) received eptacog alfa or activated prothrombin complex concentrate (aPCC), while in four patients the drugs were administered sequentially due to insufficient response or lack of availability. No patient had fatal bleeding or thrombotic events. Eight patients achieved complete remission with corticosteroids alone (53%), but two or more agents were used for four and three patients: cyclophosphamide or azathioprine was added subsequently in combination with prednisone in two patients each, while rituximab was added in two cases as early second‐line treatment following immunosuppressive combination. The laboratory changes of FVIII, inhibitor levels and the time to eradication of each patient are shown in Figure 1. After a median period of 28 days (range 10–210 days), complete remission with normal FVIII and anti‐FVIII inhibitor eradication was achieved in all but one patient, who is still on steroids 2 years after diagnosis due to inhibitor persistence (Figure 2). Given the use of immunosuppressive drugs, most patients were generally advised against breastfeeding in order to prevent the potential development of neonatal adverse events.

**FIGURE 3:**
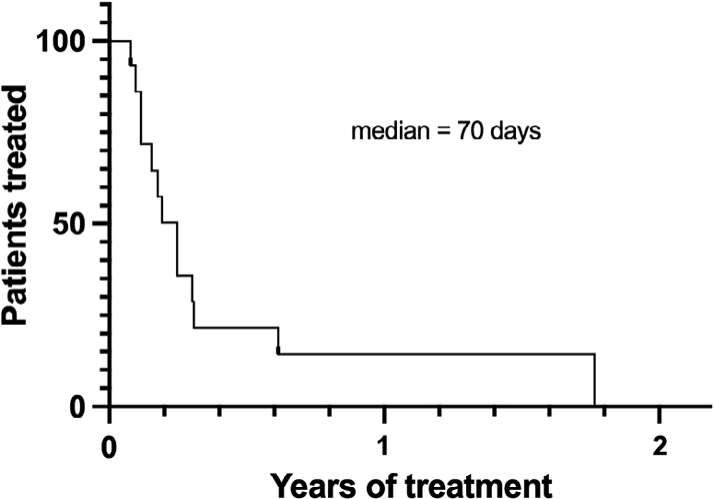
Duration of immunosuppressive treatment in women with post‐partum AHA (*n *= 15).

**FIGURE 4:**
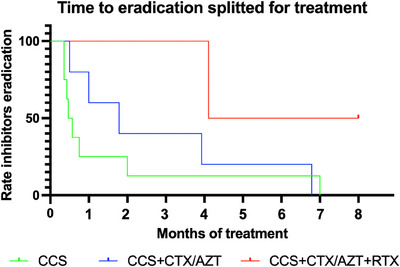
Time to eradication splitted for treatment in women with AHA (*n* = 15). CCS, corticosteroids; CTX, cyclophosphamide; AZT, azathioprine; RTX, rituximab.

However, immunosuppressive therapy, including its tapering, had a median duration of 70 days (range 28–690 days) due to high relapse risk (Figure 3). Notably, four patients required long‐term immunosuppressive therapy (more than 6 months) (Table 2). At the time of final data censoring, with a median follow‐up of 24 months, all patients were alive and well, with no significant adverse events except for a patient who experienced AHA relapse 3 months following the first episode and was successfully treated with rituximab. Around 25% of the patients reported hypertension and transient hyperglycemia as side effects of prolonged corticosteroid therapy. Splitting patients according to the type of treatment, the median time for inhibitor eradication was 14.5 days for patients treated only with corticosteroids as first‐line, 50 days for patients who required corticosteroids plus immunosuppressive drugs, and 169.5 days in two cases who needed rituximab (Figure 4).

**TABLE 2:** Treatment lines and response of patients with AHA.

	*N* patients (%)
Anti‐haemorrhagic therapy	
rFVIIa	9 (60)
aPCC	2 (13)
rFVIIa and aPCC	4 (27)
Immunosuppressive drug	
Corticosteroids (CCS)	8 (53)
CCS + Cyclophosphamide (CTX)/Azathioprine (AZA)	4 (27)
CCS + CTX + Rituximab ± AZA	2 (13)
CCS + Immunoglobulins i.v. + AZA	1 (7)
Time of immunosuppressive therapy, days (range)	70 (28–690)
Short‐term immunosuppressive therapy (<6 months)	11 (73)
Long‐term immunosuppressive therapy (>6 months)	4 (27)
Median time to FVIII inhibitor eradication, days	28 (10–210)
>30 days	8 (53)
<30 days	7 (47)
Complete remission	14 (93)

Abbreviations: aPCC, activated prothrombin complex concentrates; AZA, azathioprine; CCS, corticosteroids; CTX, cyclophosphamide; i.v., intravenous therapy; rFVIIa, recombinant activated factor VII.

## 4 Discussion

Most cases of AHA are idiopathic (44%–52%), but the disease has a bimodal distribution. In elderly male patients AHA is frequently associated with malignancy (6%–18%) and autoimmune disorders (9%–17%, most commonly rheumatoid arthritis), while in young women the principal risk factor is pregnancy, and the majority of reported peri/post‐partum AHA cases are diagnosed after delivery. In our series five patients, representing 33% of total study cohort, had a coexisting autoimmune disease and one additional patient had a positive autoimmune serology. They received immunosuppression with corticosteroids and, in two cases, another immunosuppressant was used in combination, respectively cyclophosphamide and azathioprine. These group of patients could potentially be considered at higher risk for pregnancy‐related immune disorders due to the pre‐existing immune system impairment. Notably, high incidence of pre‐existing autoimmune disorders was not previously reported in the other real‐world evidence studies and registries about AHA. The evidence of this data is limited by the small cohort of patients included in this series; however, pre‐existing autoimmune disorders should be considered as a possible risk factor for pregnancy‐related AHA.

All patients included in the present study developed abnormal bleeding following delivery.

In the present report, symptoms did arise after a mean period of 41 days following delivery. None of the patients reported abnormal bleeding during pregnancy, the diagnosis of AHA being triggered by abnormal bleeding postpartum. Two‐thirds of them developed major bleeding but no fatal bleeding occurred upon using by‐passing agents. Postpartum AHA has been described in the literature with a better overall survival than in patients of older age and malignancy: the death rate is less than 5% in postpartum AHA but up to 30% in the overall AHA population. This difference may be explained by the younger age and fewer comorbidities in cases with postpartum AHA.

The delayed diagnosis of acquired haemophilia poses a significant risk not only due to the potential for severe and life‐threatening haemorrhages but also because of the risk associated with performing invasive procedures in an attempt to stop the bleeding. In this regard, two patients had hysterectomies in an effort to control bleeding before receiving the correct diagnosis of acquired haemophilia. These patients not only underwent a likely unnecessary radical surgical procedure but were also exposed to the risk of uncontrolled bleeding associated with the surgery itself.

The autoantibodies implicated with AHA are often IgG antibodies that can potentially cross the placenta. This event could lead to the inhibition of foetal or newborn FVIII, resulting in the higher bleeding risk of neonates. In our case series, although the timing of autoantibody development cannot be certain and could have potentially been during pregnancy, the first bleeding events in most of them were at least 1 month after the delivery, and no bleeding events were described in the newborns of postpartum AHA patients.

Nearly all patients in our series achieved inhibitor eradication after a median of 28 days. Corticosteroids alone were sufficient for inhibitor eradication in half of the patients; cyclophosphamide and azathioprine were used in two patients each, while 20% received three or more treatment lines, using mainly rituximab. However, given that at least one immunosuppressive drug was administered in association with corticosteroids in seven of them, along with the high relapse risk in case of fast tapering, the median duration of immunosuppressive therapy was more than 2 months, and around 25% of the cases received treatment for more than 6 months. One patient never achieved inhibitor eradication and is still on immunosuppressive treatment 1 year following the disease diagnosis, while the other one had an early relapse that prompted treatment, as mentioned in Figures 2 and 3. In AHA unrelated to pregnancy, the combination of corticosteroids and cyclophosphamide emerged as one of the possible first‐line treatments, along with corticosteroids alone. The EACH2 Registry documented a higher rate of stable complete remission in patients treated with the combination of corticosteroids and cyclophosphamide compared to patients treated with corticosteroids alone [9]. There is a special concern about the use of cytotoxic drugs in breastfeeding women or during pregnancy. Nowadays, this class of drugs is no longer recommended due to the possible side effects on the baby or foetus. Thus, other possible drugs should be considered in combination with steroids. For this purpose, one possible alternative is the anti‐CD20 monoclonal antibody rituximab. Since 2006, reports and data from the registry evaluated the use of rituximab in AHA [10]. Recently one randomised clinical trial on rituximab for inhibitor eradication in AHA patients has been published, showing the non‐inferiority of the rituximab in combination with corticosteroids compared to the combination of cyclophosphamide and corticosteroids [11]. Rituximab use during pregnancy and post‐partum in several autoimmune disorders was evaluated in different studies during the last 20 years. Some of these studies reported transient B‐cell depletion, with a consequent increased risk of infections, in infants but no severe sequelae were reported. Therefore, given the potential risk of both transmission of autoantibodies and the development of adverse events in newborns, the patients in our cohort were generally advised against breastfeeding during immunosuppressive treatment.

The largest dataset on pregnancy‐related AHA from the EACH2 registry demonstrated CR rates in 75% of the population with corticosteroids alone. Unfortunately, the short length of follow‐up (406 days) did not allow us to observe long‐term relapses in women with a stable CR or to evaluate recurrence of AHA in subsequent pregnancies [12]. Furthermore, an important clinical issue regarding pregnancy‐related inhibitors is their possible recurrence in subsequent pregnancies. Although most series have found that inhibitors tend not to recur in those who attained CR, in one study, AHA recurred in four of six subsequent pregnancies in three patients [13].

In this study, we reported the use of conventionally accepted haemostatic treatment for AHA, in particular rFVIIa and aPCC. Four patients received both treatments sequentially, due to insufficient response of one of them followed by a switch to the second one, or lack of drug availability in the local pharmacy. All bleeding events were resolved successfully, therefore there was no need to consider the combined use of the above‐mentioned drugs, which would have imposed a high risk of thrombosis.

During the last decade, several reports and randomised clinical trials evaluated the efficacy and safety of recombinant porcine FVIII (rpFVIII) and emicizumab. Both drugs appear to be safe, particularly in terms of thrombotic risk, which is typically associated with other haemostatic agents. Data about emicizumab demonstrated its efficacy in the prevention of bleeding, although only inside clinical trials. Some reports also evaluated the prophylactic use of emicizumab in patients with AHA, but a larger population as part of randomised clinical trials and longer follow‐ups are required to confirm its use, especially in this extremely delicate setting of patients, before official approval by drug agencies. On the contrary, rpFVIII has already been broadly used, demonstrating its anti‐haemorrhagic efficacy, with the additional advantage of monitoring factor level with specific kits. None of the patients in our case series was treated with rpFVIII because there is no available data during pregnancy and breast‐feeding, but its use could be taken into consideration as a haemostatic agent in pregnancy‐related AHA in the future.

Dewarrat et al. published a comprehensive review that brought together pregnancy and postpartum AHA cases published up to 2021 (total 176 cases). Patients had a median age of 29.9 years and 69% were primiparal. Haemostatic treatment was necessary for 57%, while immunosuppression was implemented in 91%: mostly with steroids (85%) alone or with other immunosuppressants (27%). Rituximab was used most frequently as a second‐line treatment. The median time to CR was 12 months with steroids and 8 months with steroids associated with other immunosuppressive drugs Spontaneous remissions were described in six cases. A relapse rate of 29.4% was reported in patients with partial or complete response. Only 13% of women had second and subsequent pregnancies, and 22% developed a new episode [3].

The potency of the inhibitor is rather low in the majority of reported peri/postpartum cases [median titer ≃20 BU]. This may explain why the natural history of pregnancy‐associated AH is often characterised by a high overall survival (OS) and a higher rate of spontaneous remissions despite requiring a longer time to fully remit. In our series, the median inhibitor titer level was 35 BU, up to 156 BU in one patient, which could perhaps be explained by a higher incidence of autoimmune disease and a longer time to inhibitor eradication compared to other studies (Table 3).

**TABLE 3:** Postpartum AHA cases present in the literature from multicentre registries and real‐life experiences.

Author/Reference	Giuffrida et al. (2024)	EACH2 Registry Tengborn (2012) [12]	AICE Baudo (2003) [14]	SACHA registry Borg J.Y. (2013) [15]	Schep et al. (2020) [16]	Dedeken et al. (2009) [17]	Saxena R. et al. (2000) [18]	Ejaz et al. (2023) [19]
*N*° postpartum AHA	15	42 31 1° preg. 7 2° preg. 2 3° preg. 2 4° preg.	20 16 1° preg. 4 2° preg. (or more)	6	6	4	3	3
Median age (y)	31	34	30	NA	NA	30.5	31.3	32
Median FVIII (%)	4.4	2.5	NA	2.0	NA	0.05	<1	<0.01
Median inhibitor (BU/mL)	35	7.8	8.5	16.0	NA	3.2–272	10.6	7.2
Time (d/w/m) of bleeding after delivery	41d	89d	60d	94.5d	NA	1° 4m 2° 2m 3° 6m 4° 9d	NA	6d
Site of bleeding	9 vaginal‐uterine 5 cutaneous 1 muscle	19 subcutaneous 18 mucosal 14 musculoskeletal 2 haemarthrosis	3 vaginal‐uterine 1 C‐section 14 subcutaneous 1 musculoskeletal 1 tooth extraction	NA	NA	3 subcutaneous 1 haemorrhagic shock	1° skin/muscle 2° skin/muscle 3° hypovolemic shock	cutaneous, haematuria
Haemostatic therapy	9 rFVIIa 2 aPCC 3 rFVIIa+aPCC	12 rFVIIa 5 aPCC 1 FVIII concentrate 1 DDAVP 2 antifibrinolytics 1 NS	11 h/pFVIII /rFVIIa/ IVIg/aPCC	NA	No therapy	1° pFVIII 2° rFVIIa + pFVIII 3°NA 4°NA	1°NS 2°NS 3°FFP+aPCC	1° no therapy 2° no therapy 3° rFVIIa
IST	8 CCS 4 CCS+CTX/ AZT 2 CCS+CTX+ RTX 1 CS+IVIg	27 CCS 6 CCS+cytotoxix ag. 4 CCS+IVIg 2 CCS+RTX 3 NS	10 CCS 8 CCS+CTX/AZT/IVIg	3 IVIg alone 2 IVIg+CCS 1 NS	4 steroids 1 RTX+CCS/CTX	1°CCS+IVIg+CNIs 2°CCS+IVIg+RTX 3°CS+RTX 4°CCS+IVIg+RTX	1°CVP 2°CCS 3°CCS+CTX	1°CCS 2°CCS+RTX 3°CCS+RTX+MMF
Median IST duration, days (range)	70 (28–690)	96 (56–248)	180	NA				
Outcome	14 CR (93%) 1 PR (7%)	36 CR (86%) 2 relapse	14 1°CR (70%) 3 1°PR (15%) 1 failure (5%) 6 relapse (30%) 6 2°CR (100%)	6 CR (100%)	6 CR (100%)	4 CR (100%)	3 CR (100%)	3 CR (100%)

Abbreviations: ag., agent; AHA, acquired haemophilia A; aPCC, activated prothrombine complex concentrate; AZT, azathioprine; BU, Bethesda Unit; CCS, corticosteroids; CNIs, calcineurin inhibitors; CR, complete response; CTX, cyclophosphamide; CVP, cyclophosphamide, vincristine, prednisone; DDAVP, desmopressin; FFP, fresh frozen plasma; FVIII, factor VIII; hFVIII, human Factor VIII; IST, immunosuppressive treatment; IVIg, intra‐venous immunoglobulins; m, month; MMF, mycophenolate mofetil; NA, not assessed; NS, not specified; pFVIII, porcine Factor VIII; PR, partial response; preg., pregnancy; rFVIIa, recombinant factor VII activated; RTX, rituximab; w, week; y, year.

Apart from the European registry (EACH2), we summarised the data of national registries from Italy, France and the Netherlands in Table 3, including postpartum AHA patients.

Our data confirm that the majority of the patients with pregnancy‐related AHA have a good response to corticosteroids alone. However, in a significant group of cases, two or more immunosuppressive drugs were required to obtain inhibitor eradication. All patients included in this report were started on immunosuppressive treatment at the time of diagnosis. However, considering data reported in the literature about spontaneous remission, in patients who have minor and easily controlled bleeding and low titer inhibitor a possible option could be to withhold immunosuppression and evaluate eventual spontaneous remission. This approach could be justified based on the possible adverse effects of these medications. In our experience inhibitor was not eradicated after 30 days of immunosuppressive therapy in half of the study population and long‐term IST of at least 6 months, including tapering, was necessary in four patients (27%) due to delayed complete response. It should also be highlighted that none of the patients treated with long‐term immunosuppressive therapy experienced relapse. Therefore, the choice of immediate corticosteroid treatment or watch‐and‐wait strategy should be evaluated on a case‐to‐case basis specifically in terms of bleeding manifestations. In any case, one of the goals in managing patients with pregnancy‐related acquired haemophilia A is to minimise exposure to immunosuppressive drugs, thus reducing the risk of adverse or side effects.

The study has some limitations, mainly due to retrospective data collection and a low number of postpartum AHA patients. Time from symptoms onset to definitive diagnosis is not available. More than half of the patients were not referred to the haematologic specialist anymore after the obtainment of a complete response and for that reason the median duration of response cannot be reported. Furthermore, the treatment reported may not completely reflect the current clinical practice, particularly in regards to the availability of rpFVIII as an alternative to first‐line haemostatic agent and rituximab being part of immunosuppressive therapy, especially in case of lack of response to first‐line treatment including corticosteroids alone or in combination with cyclophosphamide or azathioprine. However, given the rarity of the disease itself and especially in pregnant women, as it can be seen in Table 3, according to our knowledge, this one represents the third largest population in the literature, thus expanding data availability regarding immunosuppressive management, improvement of treatment outcomes and disease awareness.

## 5 Conclusion

Post‐partum AHA patients are frequently described with moderate bleeding episodes at presentation, spontaneous inhibitor remission in some cases and overall good response to corticosteroids. In our population two thirds of the patients presented with major bleeding, with excellent response and complete resolution of symptoms following treatment with bypassing agents without fatal bleeding or thrombotic events. On the other hand, although all but one patient achieved complete remission with inhibitor eradication after approximately 28 days, the median duration of IST was more than 2 months. Interestingly around one‐third of the population had autoimmune disease, thus potentially increasing the risk of AHA development. Nearly half of the cases received at least one immunosuppressive drug together with corticosteroids, while approximately one‐fifth of them received three or more lines of immunosuppressive treatment, including tapering for more than 6 months. Besides the generally excellent outcome of post‐partum AHA, this data suggests that there is a subgroup of patients requiring a prolonged immunosuppressive treatment, often with two or more agents to obtain inhibitor eradication and disease resolution.

## Author Contributions

Collection and analysis of data, writing: Gabriele Sapuppo, Uros Markovic, Andrea Duminuco and Gabriella Santuccio. Collection of data and revision of the paper: Mariasanta Napolitano and Gianluca Sottilotta. Collection of data and writing: Manlio Fazio, Stephanie Grasso, Sara Frazzetto, Giuliana Giunta, Lara Gullo and Chiara Sorbello. Coagulation assays: Salvo La Penta. Supervision: Francesco Di Raimondo. Conceptualization, supervision, revision of the paper: Gaetano Giuffrida.

## Ethics Statement

This study was conducted in accordance with the International Conference on Harmonization Guidelines on Good Clinical Practice and the principles of the Declaration of Helsinki.

## Consent

Informed consent were obtained from the patients.

## Conflicts of Interest

The authors declare no conflicts of interest.

## Data Availability

Data are contained within this article.
